# A Preliminary Study of FTIR Spectroscopy as a Potential Non-Invasive Screening Tool for Pediatric Precursor B Lymphoblastic Leukemia

**DOI:** 10.3390/molecules26041174

**Published:** 2021-02-22

**Authors:** Radosław Chaber, Aneta Kowal, Paweł Jakubczyk, Christopher Arthur, Kornelia Łach, Renata Wojnarowska-Nowak, Krzysztof Kusz, Izabela Zawlik, Sylwia Paszek, Józef Cebulski

**Affiliations:** 1 Department of Pediatrics, Institute of Medical Sciences, Medical College, University of Rzeszow, Warzywna 1A, 35-310 Rzeszow, Poland; kornelia_lach@wp.pl; 2 Clinic of Pediatric Oncology and Hematology, State Hospital 2 in Rzeszow, Lwowska 60, 35-301 Rzeszow, Poland; 3 Doctoral School, Institute of Medical Sciences, Medical College, University of Rzeszow, al. T. Rejtana 16c, 35-959 Rzeszow, Poland; anet.kow@wp.pl; 4 Institute of Physics, College of Natural Sciences, University of Rzeszow, Pigonia 1, 35-310 Rzeszow, Poland; pjakub@ur.edu.pl (P.J.); wojnarowska.renata@gmail.com (R.W.-N.); kkusz96@gmail.com (K.K.); cebulski@ur.edu.pl (J.C.); 5 School of Chemistry, University of Bristol, Cantock’s Close, Bristol BS8 1TS, UK; chris.arthur@bristol.ac.uk; 6 Center for Innovative Research in Medical and Natural Sciences, University of Rzeszow, Warzywna 1A, 35-310 Rzeszow, Poland; izazawlik@gmail.com (I.Z.); sylwia.paszek@wp.pl (S.P.)

**Keywords:** acute lymphoblastic leukemia, cancer screening, spectroscopy FTIR, Lissajous curves

## Abstract

Early detection of the most common pediatric neoplasm, B-cell precursor lymphoblastic leukemia (BCP-ALL), is challenging and requires invasive bone marrow biopsies. The purpose of this study was to establish new biomarkers for early screening to detect pediatric leukemia. In this small cohort study, Fourier transform infrared (FTIR) spectra were obtained from blood sera of 10 patients with BCP-ALL and were compared with the control samples from 10 children with some conditions other than neoplasm. Using various analytical approaches, including a new physical model, some significant differences were observable. The most important include: the different peak area ratio 2965/1645 cm^−1^ (*p* = 0.002); the lower average percentage of both β-sheet and β-turn protein structures in the sera of BCP-ALL patients (*p* = 0.03); an AdaBoost-based predictive model for classifying healthy vs. BCP-ALL patients with 85% accuracy; and the phase shift of the first derivative in the spectral range 1050–1042 cm^−1^ correlating with white blood cell (WBC) and blast cell count in BCP-ALL patients contrary to the samples obtained from healthy controls. Although verification in larger groups of patients will be necessary, these promising results suggest that FTIR spectroscopy may have future potential for the early screening of BCP-ALL.

## 1. Introduction

Acute lymphoblastic leukemia (ALL) is the most frequent cancer diagnosed in children and represents approximately 25% of all cancers diagnosed up to 15 years old [1]. The annual incidence per 100,000 children is about 4–5, with a slight predominance of boys [2]. ALL arises from the malignant transformation and proliferation of lymphoid precursor cells in bone marrow, and, in children, is usually derived (80% of cases) from B-cell precursors (BCP-ALL) [3].

The diagnosis of ALL is usually established by examining bone marrow aspirates. This invasive procedure is often performed under general anesthesia in children and the indications for bone marrow biopsy should be considered carefully, particularly in younger children. Unfortunately, there are many transient conditions, such as infections, that can imitate acute leukemia in their clinical and laboratory presentation. Therefore, the implementation of an effective, rapid tool for the early detection of leukemia from serum could limit the number of unnecessary bone marrow aspirations under general anesthesia.

Fourier transform infrared spectroscopy (FTIR) is a non-destructive and label-free spectroscopic tool that can shed light on the molecular composition of samples. It provides a spectral fingerprint, usually in the mid-infrared (MIR) region (400–4000 cm^−1^), with characteristic absorbance peaks corresponding to nucleic acids, proteins, carbohydrates, and lipids. Contrary to standard diagnostic tools, FTIR is a rapid, cost-effective, and reproducible tool. Significantly, it requires minimal sample pre-processing. In recent years, many studies have demonstrated the application of FTIR spectroscopy to the early detection of cancer-specific chemical changes in tissues, cells, and biofluids, thus raising the potential that it could be used for screening and early diagnosis of neoplasms [4]. Unfortunately, few studies have thus far applied FTIR spectroscopy for cancer detection in children. We have previously shown that FTIR spectra can be helpful in pediatric Ewing sarcoma diagnosis and that it can be used as an important prognostic factor in this cancer [5,6,7,8]. To date, FTIR spectroscopy has not been extensively investigated in acute lymphoblastic leukemia, so its significance as a diagnostic tool and/or prognostic factor in ALL remains unknown.

In this paper, we report a small cohort study of the FTIR spectra of the sera from pediatric patients with suspected leukemia compared with the control sera obtained from children with conditions other than neoplasm. Any significant difference between them was further scrutinized to establish new biomarkers for early screening to detect pediatric leukemia.

## 2. Results

### 2.1. Exploratory Data Analysis

**Figure 1:**
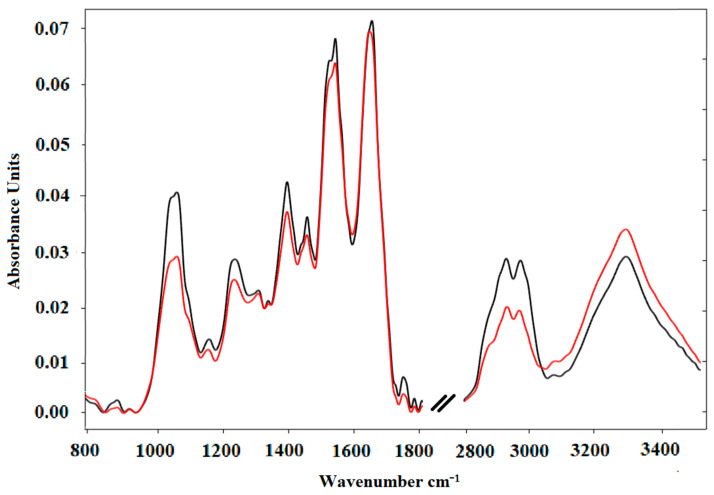
Normalized average FTIR spectra of serum samples: control (black) and Acute Lymphoblastic Leukemia Precursor B (red). Spectra cover the range of 800–3500 cm^−1^.

Figure 1 shows the average spectra of diagnosed BCP-ALL patients and healthy persons (control). The maximal absorbance and wavenumbers of the principal observed peaks along with their corresponding assigned vibrations are described in Table 1. No obvious peak shift was observed between the averaged spectra of leukemia patients and healthy individuals, though there were some differences between the intensities of some peaks. The most significant shift was observed for the peak corresponding to the amide I band (1700−1600 cm^−1^), which is due almost entirely to the C=O stretch vibrations of the peptide linkages (approximately 80%). The frequencies of the amide I band components are found to be correlated closely to the secondary structure of the proteins [9]. The position of the amide I band maximum was at 1645 cm^−1^ in the average FTIR spectrum of the control group, while the same peak maximum was shifted to 1641 cm^−1^ in the average spectrum of BCP-ALL patients.

There was no significant difference in the maximal absorbance values of representative peaks corresponding to fundamental compounds (proteins, lipids, and nucleic acids). Sheng et al. [20] have demonstrated that FTIR peak area ratios can differ between leukemia patients and healthy controls. Consequently, we took the same approach and calculated the ratios of representative peaks with one another [peaks at cm^−^^1^]: 1641/1537, 2926/1641, 2926/1537, 1070/1239, 3277/1537, 3277/1641, and 1641/1239 for the BCP-ALL group; as well as corresponding to the peak area ratios in the control group: 1645/1538, 2924/1645, 2924/1538, 1071/1241, 3278/1538, 3278/1645, and 1645/1241. As might be expected, the majority of the peak area ratios tested were not significantly different between both groups. However, the peak area ratio 2965/1645 cm^−^^1^ was significantly different, with median values for BCP-ALL vs. control of 0.54 (range 0.066–1.505) and 1.595 (range 0.585–2.527), respectively (*p* = 0.002). The peak at 2965 cm^−1^ corresponds to asymmetric stretching vibrations of CH_3_ group in lipids, while band 1645 cm^−1^ corresponds to C=O stretching of α-helix proteins. Any other differences visible in the graphs of average spectra in both BCP-ALL and controls (e.g., peaks corresponding to 1070 cm^−1^, 1241 cm^−1^, or 1396 cm^−1^) were not statistically significant and are irrelevant for classification.

### 2.2. The Secondary Structure of Proteins

**Figure 2:**
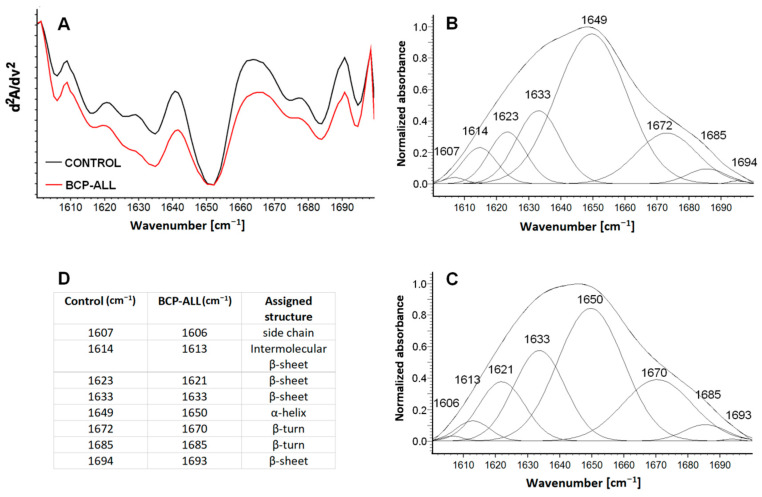
The second derivative (**A**) for the control samples (black line) and for the BCP-ALL samples (red line) and the curve-fitting analysis in the amide I region in the control samples (**B**) and in the BCP-ALL samples (**C**). Assignement of individual components to various protein secondary structures is described in table (**D**).

Figure 2 shows the d^2^A/dν^2^ normalized spectrum for the region from the 1600 cm^−1^ to the 1700 cm^−^^1^, and the Gaussian deconvolution of the amide I band for the control and BCP-ALL samples. The positions of the second derivatives minima correspond to the positions of the individual spectral lines. These lines overlap, forming the amide I band, but each of them can be assigned to a specific protein conformation [21,22,23]. The differences in protein conformation may indicate changes due to the disease process.

The position of the amide I band is similar for the control and the BCP-ALL samples; however, its shape is slightly different. The second derivative spectra (shown in Figure 2A) are very similar in terms of the lines’ composition, but some of their amplitude is noticeably different. This corresponds with the different intensity of the individual spectral lines forming the amide I band (Figure 2B,C).

FTIR allows access to bulk information on the secondary structure of the proteins present. In the average spectrum of the control samples, the line located at 1649 cm^−1^ is associated with an α-helical protein structure [9,21,23,24], and the relative area of its Gaussian provides a concentration of about 51%. The percentage of the β-sheets is half the size and is about 25%, calculated by the relative area of the Gaussian for the lines at 1623 cm^−1^, 1633 cm^−1^, 1694 cm^−1^ in the averaged spectra [21,24,25,26]. β-turns, which are a type of non-regular secondary structure that causes a change in direction of the polypeptide chain, has a 17.5% contribution. It is associated with the relative area of lines at 1672 cm^−1^ and 1685 cm^−1^ [9,23,26]. The line at 1614 cm^−1^ corresponds to the intermolecular β-sheets [25], and its contribution in the averaged spectra is about 6%. The intermolecular β-sheets are characterized by stronger hydrogen bonds [16]. The bands originating from the amino acid side chains vibrations are also observed (1607 cm^−1^) [21].

The positions of the lines observed after deconvolution of the amide I for BCP-ALL samples are very similar. The recorded shifts are at the 1–2 cm^−1^ level. Major differences relate to the surface of the registered lines and the contribution of individual protein structures. The α-helical protein structure is distinctly smaller and is about 40% (a decrease of about 11%), whereas the percentage of the β-sheets is larger (about 34%, an increase of approximately 6%). Similarly, the β-turn structure participation also increases (about 23%, an increase of approximately 5%). The proportion of intermolecular β-sheets decreased by about two times (about 3%) compared with the control sample. The same line is recorded for the amino acid side chain vibrations. The values above were calculated on the analysis of the average spectra for BCP-ALL and controls.

**Figure 3:**
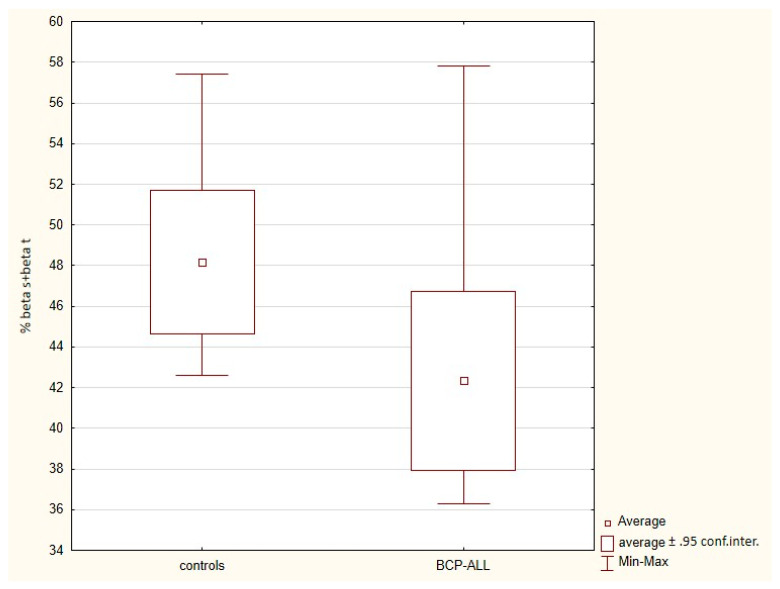
The average percentage of both β-sheet and β-turn (% βs + βt) protein structures in the sera of controls and BCP-ALL patients calculated as the average of the sum of the individual values in each sample (*p* = 0.030).

When the average percentage composition of secondary protein structure is calculated as the average of the sum of the individual values in each sample, then both β-sheet and β-turn (% βs + βt) protein structures content is significantly lower in the sera of BCP-ALL patients compared to the control group (42.34% vs. 48.19%; *p* = 0.030); see Table 2 and Figure 3.

The discrepancy between the secondary structure protein composition calculated directly from the averaged FTIR spectrum and that calculated as the average of the sum of the individual values follows from the non-linearity of biological systems.

Thus, the % βs + βt seems to be a suitable biomarker to distinguish the ALL cohort from controls. Therefore, in the next step, the cut-off values of % βs + βt were determined using receiver operating characteristic analysis (ROC) implementing the Youden index, which can differentiate BCP-ALL patients and controls with the greatest accuracy. The cut-off value 42.3 was obtained with AUC 0.82; 95% AUC 0.615–1.0; sensitivity 0.6; specificity 1.0; accuracy 0.8.

### 2.3. Methods for Dimensionality Reduction

To differentiate the FTIR serum spectra of the BCP-ALL patients and controls, we turned in the first instance to unsupervised dimensionality reduction. Dimensionality reduction approaches are broadly based on the selection of the informative features, or the generation of variables, that retain the information present in the original dataset. We analyzed the spectra by a range of matrix decomposition (including principal and independent components analysis (PCA and ICA); various kernel PCA methods; and manifold learning approaches, which included t-distributed stochastic neighbor embedding (tSNE), locally linear embedding (LLE), and isometric feature mapping (IsoMap)), as implemented in the Python library Scikit-Learn. We did not observe any clustering of the analyzed groups. When the first derivative of analyzed spectral data was taken instead, some separation of the data became possible, although still with significant overlap. It was clear though that the first derivative spectra were more discriminating for classification than the raw spectra alone.

**Figure 4:**
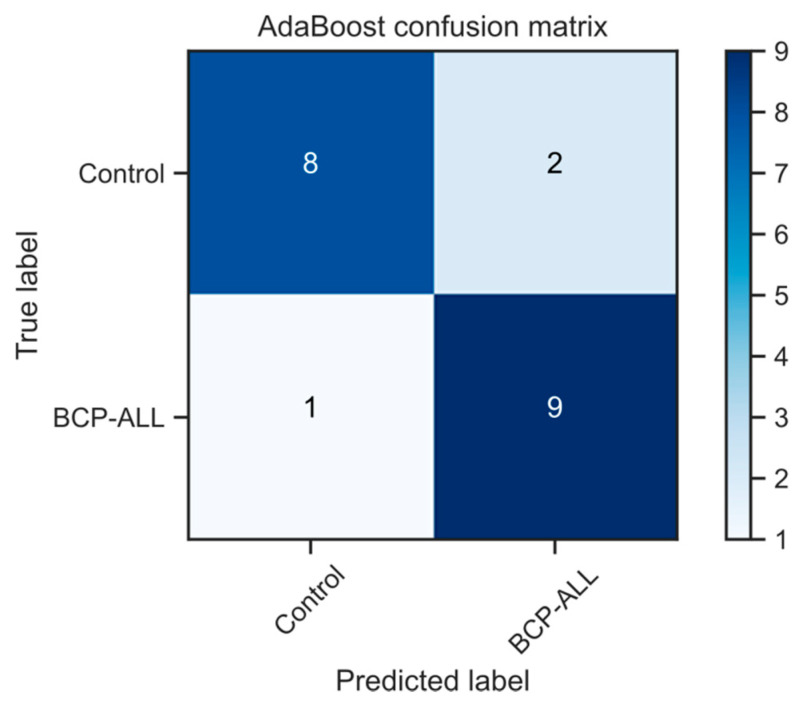
Confusion matrix of an AdaBoost prediction model based on principal components of the first derivative spectra. This model achieves an 85% accuracy as assessed by leave-one-out cross-validation.

Reducing the data to the first principal components of the first derivative spectra (see Supplementary Figures S2 and S4 for scores and loadings plots, respectively) and screening a range of classification algorithms, we were able to generate an AdaBoost-based predictive model that was capable of classifying healthy vs. BCP-ALL patients with 85% accuracy (Figure 4 shows the confusion matrix for this model using leave-one-out cross-validation). Due to the small cohort size, leave-one-out cross-validation was used for model accuracy assessment. Leave-one-out cross-validation has been shown to have low bias and low variance in tasks that contain low numbers of samples, such as in this case, and hence minimizes the risk of over-fitting.

### 2.4. The Types of Absorbance Dynamics FTIR Spectra—Lissajous Curves Construction

Based on the findings outlined above, and to focus only on dynamics of the spectra (i.e., the rate of absorbance change as a function of the wavenumber), the first derivative of the IR spectra was considered. This approach can also help eliminate variable sample thicknesses during preparation.

In the first derivative IR spectra A, for a carefully chosen range of wavenumbers k, one can distinguish two types of absorbance dynamics.

For the first type, we have $\frac{dA}{dk}>0$, where A denotes the IR spectra, and for the second type, $\frac{dA}{dk}<0$ (see Supplementary Figures S5 and S6).

Physically, this means that we have opposite changes in the absorption. In the case when $\frac{dA}{dk}>0$, the absorption is raising, whereas if $\frac{dA}{dk}<0$, the absorption is lowering in time. To show explicitly the difference between these types of dynamics, which take place in spectra obtained for leukemia and control groups, we have developed a method that is based on the technique of Lissajous curves. A Lissajous curve is the graph of a system of parametric equations which describe harmonic motion. The shape of this curve allows the determination of, among other things, the phase shift between equations. In our approach, instead of two parametric equations, we use two IR spectra, and Lissajous curves help us to determine the phase shift between these spectra in particular regions of wavenumbers. The first IR spectra is a reference spectrum (RefSpec), calculated as an average of all IR spectra obtained for healthy persons (control group), whereas the second is the IR spectra of individual patients (PatSpec) belonging to BCP-ALL group or spectra (ConSpec) of persons in the control group.

**Figure 5:**
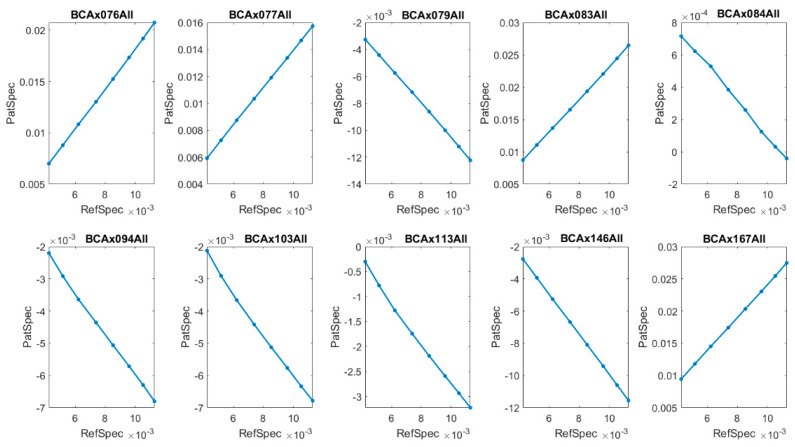
Lissajous curve for (RefSpec(k), PatSpec(k)), *k* ∈ (1042,1050) [cm^−1^]) for ten BCP-ALL patients. The phase shift between IR spectra of patients: BCAx076ALL, BCAx077ALL, BCAx083ALL, BCAx167ALL, and reference IR spectra RefSpec is equal to zero, whereas for the remaining patients it is equal to π.

**Figure 6:**
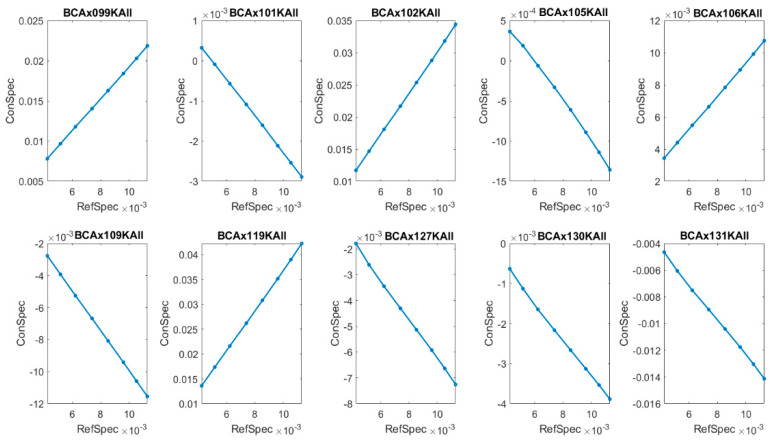
Lissajous curve for (RefSpec(k), ConSpec(k)), k∈(1042, 1050) [cm^−1^]) for the control group. The phase shift between IR spectra of patients: BCAx099KALL, BCAx102KALL, BCAx106KALL, BCAx119KALL, and reference IR spectra RefSpec is equal to zero, whereas for the remaining patients it is equal to π.

Presentation of the data on the Cartesian space ${\mathbb{R}}^{2}$ of the form (RefSpec(k), PatSpec(k)) or (RefSpec(k), ConSpec(k)), where k denotes wavenumbers in [cm^−^^1^], reveals graphical patterns which can be used to classify patients or persons from the control group, according to the absorption dynamics. Strictly speaking, the graphs (RefSpec(k), PatSpec(k)) and (RefSpec(k), ConSpec(k)) on Cartesian space for the region $k\in \left(k_{1},\;k_{2}\right)$ correspond to Lissajous curve. The shape of these curves is directly related to the phase shift between the considered IR spectra. In this way, we obtained a very sensitive method, which allows the discovery of useful markers for patient classification. Markers can be found in carefully selected narrow regions of wavenumbers:

(1) $$\left(k_{1},{\;k}_{2}\right),{\;k}_{1}<k_{2},{\;k}_{2}=k_{1}+\Delta$$

where Δ is the width of the region. As an example, in Figure 5 and Figure 6 we present markers in the region $k\in \left(1042,\;1050\right)\;\left[{\mathrm{cm}}^{-1}\right]$ for BCP-ALL patients and the control group, respectively.

In these narrow regions of wavenumbers, two opposite dynamics can be noticed for the patients, namely covariant and contravariant compared to the reference IR spectra. These dynamics can be labelled by phase shift between the first derivative of reference IR spectra (RefSpec) and the first derivative of IR spectra for patients, PatSpec or ConSpec. In the covariant case, the phase shift is equal to 0, and in the contravariant case, it is equal to π (see examples in Figure 5 and Figure 6).

This classification in terms of phase shift for leukemia patients is strictly correlated with their level of white blood count (see Table 3), and even more so, it relates to the number of circulating blast cells in peripheral blood.

A level of blast cells greater than 1.0 × 10^3^/µL and total white blood cells count greater than 9.0 × 10^3^/µL corresponds to a phase shift equal to zero, and in the opposite case, corresponds to π. For persons in the control group, no relationship with WBC level was observed (Table 4).

## 3. Discussion

The possibility of the early detection of leukemia in children based only on the analysis of their serum FTIR spectra seems an attractive tool for routine medical practice. Although acute leukemia is the most common pediatric neoplasm and one of the more frequent neoplasms in adults [27], there are few studies on the application of FTIR spectroscopy to early diagnostics, compared to other cancer entities [4].

Sheng et al. [20] showed that the ratios of particular corrected peaks heights (measured following Yano’s method) could differentiate the serum of leukemia patients from that of healthy controls. The H2959/H2931 ratio, representing the ratio of CH_3_/CH_2_ groups, had the highest significant difference. Furthermore, from curve fitting, the RNA/DNA (A1115/A1028) ratios were lower in leukemia patients’ serum. Unfortunately, the examined sera were obtained from patients with different types of leukemia (AML, 22 pts; CML, 4 pts.; ALL, 4 pts).

In another study (Erukhimovitch et al. [28]), the authors showed that peaks at 1056 cm^−1^ (corresponding to carbohydrates), 1270 cm^−1^ (amid III), and 1592 cm^−1^ (amino acids) were significantly reduced in spectra obtained from plasma of healthy persons compared to patients with chronic lymphocytic leukemia. Furthermore, cluster analysis of the obtained spectra at those specific regions provided an excellent classification of the healthy and the patient samples, which correlate completely with clinical data.

Previous publications have reported the application of FTIR to the diagnosis and monitoring of acute lymphoblastic leukemia. These studies have focused, however, on the examination of bone marrow or isolated lymphocytes examination [29,30,31,32]. To our knowledge, this is the first report of the FTIR analysis of sera obtained from a homogenous group of ALL patients for early diagnostics. We were able to show differences between leukemic and control sera at two levels. The first distinction has concerned the pattern of the whole spectrum. Moreover, there have been identified some different regions and peaks of the spectrum which could be applied to separate control and ALL patient sera.

In our initial data analysis, we found that the first derivative of the spectral data allowed for greater discrimination between the patient groups. From this, we developed a new original approach for spectral data analysis based on Lissajous figures and on the dynamics of the absorbance in spectra. The first derivative of the spectra was used to plot Lissajous figures. Their phase shift in the spectral range 1050–1042 cm^−1^ is correlated with WBC as well as blast cell count in BCP-ALL patients, contrary to the samples obtained from healthy controls, wherein no relationship with WBC was confirmed. We have shown that this connection is not random. It can be explained by different composition of leukemic sera resulting in the rapid proliferation of leukemic cells in blood and bone marrow. Compared with controls, patients with acute leukemia show serum metabonomic differences involving aberrant metabolism pathways including glycolysis, TCA cycle, lipoprotein changes, choline, and fatty acid metabolisms [33,34]. The next major difference we have shown was a significantly lower content of β-sheet and β-turn in the protein component of sera of leukemic children. This is contrary to the results obtained by G.A. Raouf et al. [32], who studied free bone marrow samples, which showed that there was a relatively high proportion of anti-parallel β-sheet protein in ALL patients. This difference may arise from the nature of the analyzed tissue (serum vs. bone marrow cells). The accuracy of the test based solely on the β-sheet and β-turn protein content in serum was about 80%. Finally, we have found some detailed differences concerning the single peaks in the spectra, like the ratio of peaks at 2965 cm^−1^ and 1645 cm^−1^ (2965/1645) and the position of the peak for the amide I band maximum in the average leukemic and normal FTIR spectrum.

We are conscious that the number of analyzed samples is too small to draw definitive and strong conclusions about the clinical importance and practical application of the obtained results. We believe though that these results are promising and that they justify further studies in larger groups of patients. After positive verification, this tool could be applied for early ALL screening.

In conclusion, there are some interesting differences between the FTIR spectral profile of leukemic and normal serum. These differences may offer a potential route to the early identification of children with ALL using FTIR spectroscopy and in so doing could limit the number of invasive procedures and accelerate the diagnosis of individuals. These results must be verified in prospective studies in larger groups of patients and healthy individuals.

## 4. Materials and Methods

### 4.1. Patients

Ten patients with newly diagnosed BCP-ALL were included in this study. They all were hospitalized in the Department of Pediatric Hemato-oncology, Clinical Regional Hospital in Rzeszow, Poland. The median age of our study group was 8 years (range: 2–17 years.), and the male/female ratio was 3:2. All serum samples were obtained at diagnosis for routine medical tests. The diagnosis was confirmed by bone marrow biopsy with the expression of the antigens corresponding to precursor B lymphocytes.

Four healthy pediatric donors and six children with conditions other than neoplasm and benign conditions were included in the control group. All blood samples were taken because of other medical indications. The median age in this group was 8 years. (range: 0.5–15.5 years), and the male/female ratio was 1:1.

The study was conducted under Institutional Review Board Protocol No. 1/01/2020 from 30/01/2020 at the University of Rzeszow. The experimental protocols used in this study were approved by the institutional ethics committees (IECs) of the University of Rzeszow and were carried out following the approved guidelines. Informed consent was obtained from all subjects or their guardians before blood sample collection.

### 4.2. Sample Preparation

Following standard procedures, whole blood samples were collected into clot activator tubes and were left to clot at room temperature for a minimum of 30 min and a maximum of 2 h. Blood serum was obtained by two-step centrifugation; first at 3000 rcf for 5 min, and then the supernatant from this was recentrifuged (5000 rpm for 5 min) to prevent blood cells contaminating the FTIR spectrum. All serum samples were frozen (−80 °C) until analysis.

### 4.3. FTIR Spectroscopy

Shortly before analysis, serum samples were thawed at room temperature and 10 µL of blood serum was pipetted onto the calcium fluoride (CaF_2_) slides and left to dry for approximately one hour to eliminate water interference in FTIR spectra.

All sera spectra were acquired in the mid-infrared (MIR) range of 400–4000 cm^−1^, with a spectral resolution of 2 cm^−1^, and are the average of 64 scans without air compensation, using a Bruker Vertex 70v FTIR spectrometer (Bruker, Poznan, Poland) equipped with attenuated total reflection (ATR) plate, single-reflection snap ATR crystal as a source of mid-infrared radiation, and MCT (Mercury–Cadmium–Telluride) IR (infrared) detector.

The plate was cleaned with ethanol (95%) before each spectrum was recorded and the air was measured as a background. For each serum sample spectra were recorded in duplicate or triplicate. A total of 63 spectra were collected during this study.

### 4.4. The Secondary Structure of Proteins

To investigate the secondary structure of proteins contained in the tested serum samples, the second derivative analysis and the curve-fitting procedure in the amide I spectral region was applied. The lines forming this band are highly sensitive to molecular geometry and hydrogen bonding, which allows for the analysis of the protein secondary structure [9,21,35]. The analysis of protein secondary structure was performed by studying the contribution of the individual lines composing the amide I band (1600–1700 cm^−^^1^). In the first step, the analysis of the second derivative for a given region of individual spectra was calculated using the Savitzky–Golay differentiation (baseline correction: Y = 0; differentiation order, 2; window size, 21; polynomial order, 7). The obtained information was used for the curve-fitting procedure. Gaussian functions were then fitted to the observed bands. The analyzed band consists of 8 spectral lines. The sum of the value of all maxima absorbance corresponding to α-helix and β-sheet were considered.

### 4.5. Data Analysis

For all obtained spectra, vector normalization and baseline correction were applied. These operations were performed using OPUS 7.0 (provided by Bruker Optik GmbH/version 7.0, 2011, https://opus-application.software.informer.com/7.0/) and KnowItAll Academic Edition (John Wiley & Sons, Inc., version 2018, https://sciencesolutions.wiley.com/academic-edition/). Moreover, in each FTIR spectrum, vibrations corresponding to nucleic acid, phospholipids, proteins, and lipids were analyzed. The number of obtained data was from FTIR; therefore, to determine a similarity between analyzed groups, a PCA analysis was done. PCA reduces the dimensionality, the number of variables of the data, by maintaining as much variance as possible. This analysis was done using Past software (version 4.04, https://www.nhm.uio.no/english/research/infrastructure/past/). Moreover, to determine the similarity between samples within the groups, a hierarchical cluster analysis (HCA), using Past software, was done. Further data analysis including clustering and dimensionality reduction was performed using Python 3.6 (Python Software Foundation, version 3.6, https://www.python.org/downloads/) and Scikit Learn 0.19.1 (BSD License, version 0.19.1, https://pypi.org/project/scikit-learn/0.19.1/).

The *t*-test was used to determine the statistical significance of the difference between two sets of data with a normal distribution.

The optimal cut-off points for distinguishing BCP-ALL vs. the control group using an average α-helix percentage/β-sheet + β-turn percentage were determined using receiver operating characteristic analysis (ROC) by implementing the Youden index.

The level of significance was *p* < 0.05. The calculations were performed using Dell Inc.’s Dell Statistica (data analysis software system), version 13 (2016).

## Figures and Tables

**Table 1:** FTIR peaks position with corresponding vibrations in the analyzed three groups.

FTIR Spectroscopy Peaks				
Control Sample		ALL Sample		ASSIGNMENT
Peaks [cm^−1^]	Average [A.U.]	Peaks [cm^−1^]	Average [A.U.]	
894	0.002	893	0.001	C-C, C-O deoxyribose, fatty acid, saccharide [10]
931	0.001	932	0.001	Left-handed helix DNA (Z form) [11]
1071	0.039	1070	0.029	v_s_(PO_2_^−^) DNA, RNA phospholipid, phosphorylated protein [12]
1166	0.013	1163	0.012	C-O group from groups of serine, threonine, and tyrosine of protein [10]
1241	0.027	1239	0.025	PO_2_^−^ asymmetric and symmetric stretching (nucleic acids, phosphorylated proteins, and phospholipids) [13,14]
1311	0.022	1311	0.022	Amide III: proteins [15]
1340	0.02	1340	0.02	CH_3_, CH_2_ wagging: lipids/proteins [16]
1396	0.041	1396	0.037	ν_s_(CH_3_): proteins, COO- symmetric stretching: fatty acids [15,16]
1455	0.034	1454	0.033	CH_3_, CH_2_ bending modes: lipids/proteins [16]
1538	0.066	1537	0.064	Amide II due to N-H bending of proteins [15,17]
1645	0.07	1641	0.071	Amide I due to C=O stretching of α-helix proteins [17]
2924	0.028	2926	0.02	asymmetric stretching symmetric CH_2_: lipids [18]
2965	0.027	2962	0.019	asymmetric stretching vibrations of CH_3_ [17]
3278	0.028	3277	0.034	ʋ-NH stretching of the peptide bond (-NHCO) of proteins and ʋ-OH stretching of functional groups of water [19]

**Table 2:** The secondary structure composition (%) for the control and BCP-ALL average samples. They were calculated as the average of the sum of the individual values in each sample.

Sample	Secondary Structure		
	α-Helix (%)	β-Sheet + β-Turn (%)	Other Structure (%)
Control	44.55	48.19	7.26
BCP-ALL	50.42	42.34	7.24

**Table 3:** Correlations between phase shift and WBC and blast cell counts in peripheral blood for BCP-ALL patients.

Patients	BCAx076 ALL	BCAx077 ALL	BCAx079 ALL	BCAx083 ALL	BCAx084 ALL	BCAx094 ALL	BCAx103 ALL	BCAx113 ALL	BCAx146 ALL	BCAx167 ALL
Phase Shift	0	0	π	0	π	π	π	π	π	0
WBC [10^3^/µL]	205.8	21.08	1.7	9.08	3.33	2.22	0.13	1.62	4.15	22.43
Blast Cells in Peripheral Blood [10^3^/µL]	183.16	11.80	0.24	2.81	0.66	0	0	0.19	0.33	10.99

**Table 4:** Correlations between phase shift and WBC level for the control group.

Controls	BCAx099KAll	BCAx101KAll	BCAx102KAll	BCAx105KAll	BCAx106KAll	BCAx109KAll	BCAx119KAll	BCAx127KAll	BCAx130KAll	BCAx131KAll
Phase Shift	0	π	0	π	0	π	0	π	π	π
WBC [10^3^/µL]	3.98	7.38	4.86	5.37	10.63	5.61	10.50	7.99	3.6	5.24

## Data Availability

The data presented in this study is available in Supplementary Materials.

## Supplementary Materials

The following are available online: Supplementary Figures S1–S6, Supplementary Excel File 1.

## Sample Availability

Not available.
